# Safe Navigation Distance Between Marine Routes and Aquaculture Farms in South Korea Using Gaussian Mixture Model

**DOI:** 10.3390/s20051246

**Published:** 2020-02-25

**Authors:** Sang-Lok Yoo, Jong-Chul Jeong

**Affiliations:** 1 Department of Computer Science, Chungbuk National University, Cheongju 28644, Korea; sanglokyoo@cbnu.ac.kr; 2 Department of GIS Engineering, Namseoul University, Cheonan 31020, Korea

**Keywords:** aquaculture farms, separation distance, traffic route, satellite and aerial photographs, Gaussian mixture model, IoUT (Internet of Underwater Things), sensor

## Abstract

The purpose of this study was to determine the minimum separation distance between aquaculture farms and ship traffic to prevent damage to either the farms or the vessels. A high-risk area in South Korea was selected for the study by overlapping shipping routes with fisheries using satellite and aerial photographs. The annual frequency of damage was calculated based on a probability distribution applied to the sea area, and a safe distance between the aquaculture farms and the traffic was derived. The Kolmogorov–Smirnov (KS) test was conducted to determine whether the Gaussian mixture model (GMM) follows the data of this study. It was found that a safe distance of at least 1000 m is needed to avoid farm or vessel damage. Then, it is possible to prevent damage to vessel propellers and fisheries locating aquaculture farms at the minimum safe distance from the traffic routes. For protection and security of these structures, the installation of a set of wirelessly Internet of Underwater Things (IoUT) sensors that can transmit the farm location to the ship’s navigator were suggested.

## 1. Instruction

According to the Food and Agricultural Organization (FAO) of the United Nations, one of the most increasing food-producing industries in the world is the aquaculture activity, which is a vital industry necessary to meet the future worldwide demand of seafood [1].

Since 1970, approximately, aquaculture had an average annual growth of 8.9% and is one of the fastest developing food industries in the world [2]. In addition, Menicou et al. [3] considered that one of the most increasing industrial activities around the world was the aquaculture industrial sector. Similarly, the aquaculture production of finfish species is the main supplier of seafood that has experimented a continuos commercial and economic growing income during the last years [4].

About South Korea, the demand for high-end seafood is increasing, and the production of aquaculture farms, including those for shellfish and seaweed, is the primary source of income for fisheries, accounting for 56.4% of South Korean fisheries [5]. The total area used by aquaculture farms in South Korea is increasing every year. In particular, as of 2016, the Jeollanam-do province accounts for 74.1% of the national aquaculture farms [6]. Likewise, while the fishery population has decreased by more than 70,000 tons, the net income of aquaculture farms has increased by 2.4 times during the last 10 years.

However, owing to the rising number of aquaculture farms, the damage caused to farms by vessels is growing, and the morale of fishers is decreasing due to damages costing more than several million dollars per incident. In addition, accidents in aquaculture farms may lead to secondary problems such as vessel propeller failure accidents and damage to the property of fishers [6]. Therefore, the placement of aquaculture farms is essential in ensuring the operational efficiency of fisheries, the protection of farm property, and the safe navigation of vessels.

Regarding this issue, it is necessary to comment that the optimum location of aquaculture farms is a component of marine spatial planning (MSP) that considers the interaction between navigation channels and aquaculture structures. The adequated settings of these farms is a complex problem that require a coordinated and integrated management vision that assure the organization, regulation, zoning, security, protection, and sustainability of the aquaculture farms on the sea and coasts [7,8,9].

On the other hand, Maritime New Zealand [10] provides guidelines for establishing aquaculture farms in New Zealand. According to the guidelines, offshore aquaculture farms shall not be located within 1000 m of any recognized navigational traffic route, and inshore aquaculture farms shall not be placed within 500 m of any known navigational circulation path. However, these criteria do not provide any statistical basis to address the above mentioned issues. The United Arab Emirates (UAE) Aquaculture Guide, states that farming sites should be located at a ‘safe’ distance from a navigational path [11]. Yet, specific distance measures were not provided. The International Association of Marine Aids to Navigation and Lighthouse Authorities (IALA) guidelines on navigational safety within marine spatial planning, IALA (2017), encourage the use of geographic information systems and shipping route information to analyze possible conflicts between area uses, including the distance between wind farm areas and shipping routes [12]. The guideline states that the sites of aquaculture farms should be deployed at a ‘safe’ distance from traffic routes. Nonetheless, the specific distance measures were not offered in this case as well. Gentry et al. discussed the impact of aquaculture on other marine activities including shipping [8]. The authors proposed to perform a trade-off analysis that can provide guidance on how spatial planning can be used to minimize internal conflicts. The methods of analysis that are suitable for shipping fairway and aquaculture conflicts were not explicitly stated. Istikbal and Erkan [13] presented a case study of recent vessel accidents that involved aquaculture farms around Turkey. The authors proposed the use of pilotage services to protect the marine environment in or near where the aquaculture farms are located and ensure the safety of the sea area in question. However, the suggested method cannot be applied to other aquaculture farms that are not subject to compulsory pilotage.

Regarding previous studies in related fields, Wawruch and Stupak [14] suggested a probabilistic approach for modeling the safe distance between vessel traffic routes and wind farms. This study used two collision probability models proposed by Germanischer Lloyd and Dutch Maritime Research Institute, both of which modeled the maritime traffic perpendicular to the direction of wind farms as a Normal distribution. A fitness test was not performed even though the Normal distribution was used. The limitation of this study was that the Normal distribution may not be appropriate owing to the nature of the sea, and other probability distributions that might have been more appropriate in this case were not considered. In another related study on planning the safe transit of a vessel through a mapped minefield, Bekker and Schmid [15] proposed a graph-theory-based solution where the navigable sea was considered as a grid. In this model, the graph nodes represent locations on the grid, and the edges symbolize the cost of travel between each node. The cost is modeled to consider the physical distance between the grid points and the danger associated during the journey between the grid points. While this is a robust mathematical model applicable in actual situations, the modeling of aquaculture farms in the form of point sources of danger is difficult and not feasible.

There are studies on the protection of aquaculture farms through the development of surveillance systems [16,17,18]; however, these studies have not addressed separation distances.

Furthermore, aquaculture farms are not marked on nautical charts. Thus, the risk of the accidents caused by propeller failure owing to damage to aquaculture farms is increasing for the vessels navigating the coast [6]. Indeed, it is difficult to find the exact location of aquaculture farms because they are widely distributed on the sea, and research is being conducted using satellite and aerial images to understand the distribution and status of aquaculture farms [19,20,21]. In relation to this topic, marine spatial planning (MSP) and the use of technologies such as the automatic identification systems (AIS) can contribute to the location, security, planning, and management of aquaculture farms on the sea [7,8,9,22].

It is also convenient to note that the utilization of underwater sensors to detect the distance between fishing farms and ships can be useful for their protection and safety. In this sense, we can use the concept of the Internet of Underwater Things (IoUT) to digitally observe and monitor aquaculture farms and ships distances. Currently, IoUT is a new aspect of Internet of Things (IoT) through which it is intended to generate a global network of interconnected and intelligent underwater sensors that allow digitally connecting and monitoring the seas, oceans, and lakes of all the world [23]. In relation to aquaculture farming, the possibility of using IoUT brings several advantages such as the enhanced design of aquaculture farms, better quality of sea products, costs reduction, better environmental handling, minor losses, reduced management of aquaculture farms, and security, among other possibilities [23]. About this topic, in a previous study, was designed and implemented a wireless remote sensor that monitors and evaluates digitally the environment of a fish farm [24]. In China, Zhu et al. [25] designed a remote monitoring system that used wireless communication technology to provide water-quality information for intensive aquaculture farming. In a related study, the environment of an aquaculture farm was simulated using a wireless network of sensors that monitors remotely some variables such as temperature, PH, oxygen content and water level [26]. In addition, Føre et al. [4] developed the concept of Precision Fish Farming (PFF) to monitor and enhance the biological processes of fish production using control engineering systems. In another study, the authors developed and tested in an aquaculture farm in Norway, an acoustic monitoring fish system based on the Internet of Fish (IoF) concept that provides fish telemetry data [27].

In our study, we collected the location data of aquaculture farms using satellite and aerial images in the waters close to Wando Island, which has one of the largest aquaculture farms in South Korea. Then, we calculated the lateral distance between the vessels and the closest aquaculture farms and applied the Gaussian mixture model (GMM) derived from the optimal probability distribution [28]. The separation distance between traffic routes and the aquaculture farms installed on these routes was then estimated. In this sense, the purpose of this study was to determine using statistical techniques the minimum separation distance between the aquaculture farms and the traffic routes to prevent farm property damage and vessel propeller failure. In addition, the use of a IoUT was suggested to transmit the distance information. In relation to our objective, it is pertinent to note that up to the author’s knowledge there are no published studies that estimate from a statistical point of view the minimum safe distance that should exist between aquaculture farms and navigation ships.

## 2. Materials and Methods

### 2.1. Study Area

**Figure 1:**
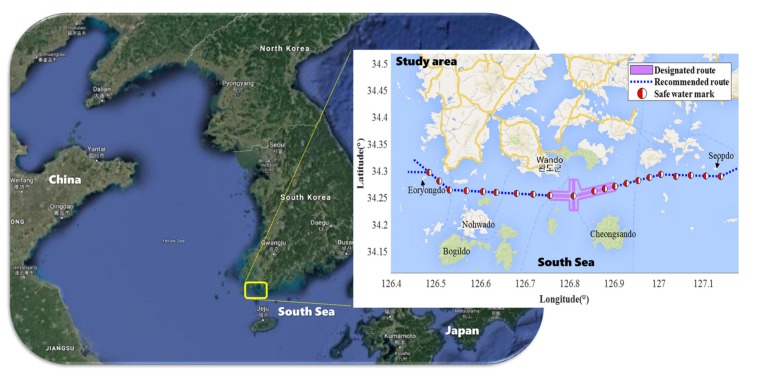
The study area.

The spatial range of this study was set in the waters near Wando, where Korean aquaculture farms are the most abundant. As shown in Figure 1, the study area covers numerous islands and a complex coastline, and it is located between latitudes 34.1° N and 34.5° N, and longitudes 126.4° E and 127.2° E. In Wando County, there are many aquaculture farms for seaweed, kelp, and for more than 80% of the domestic abalone [21].

In this figure is shown the traffic route in the waters near Wando between Eoryongdo and Seopdo for safe vessel navigation. To clarify the traffic route, there are twenty-one safe watermarks with red and white longitudinal lines in the east-west direction [28,29]. It is necessary to navigate to the starboard side of the traffic route [30]. It is specified not to deviate from the traffic route to prevent damage to the farms distributed along the starboard side of the direction of a course.

### 2.2. Map Overlay Development

**Figure 2:**
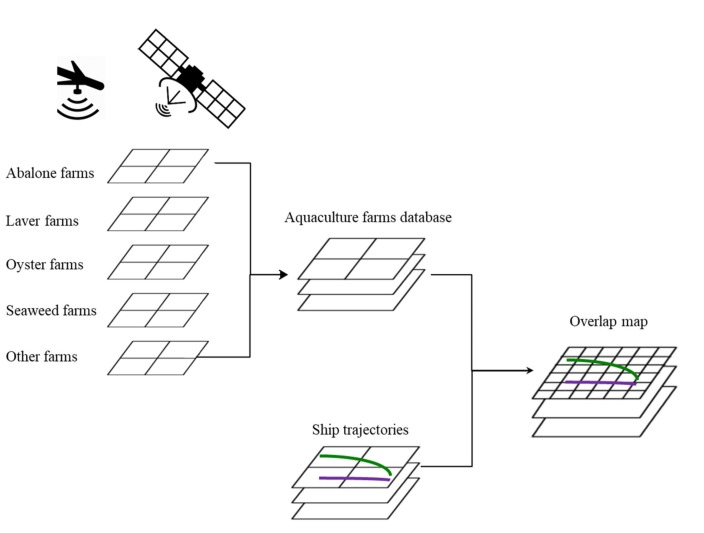
The procedure of overlapping maps.

Figure 2 shows the overlap process. First, the satellite images of the study area were processed to create a map of the locations of the aquaculture farms in the Wando region. In this procedure, we constructed an integrated aquaculture farm database after collecting the data for each kind of aquaculture farm. Second, the present study used the Automatic Identification System (AIS) data for the year 2014. The data were provided by the Ministry of Oceans and Fisheries of South Korea. The data set consisted of information on vessels, including the Maritime Mobile Service Identity (MMSI), name, breadth, length, course, position, and speed. Moreover, certain dynamic information, such as position (latitude and longitude), and the course of the vessel were selected. The AIS data was distinguished by the vessel. The data were filtered based on ranges of 2 to 7, where the number represents the MMSI, which identifies a specific vessel. The errors in AIS information regarding the vessel type [31] were corrected using the data published by the port management information system data of South Korea [32] and the International Telecommunication Union [33]. Fishing boats were excluded from this study because they work near to aquaculture farms without causing damage. After setting a gate line, only the vessels navigating along the traffic route were extracted. Then, the area for high risk of serious damage to fisheries was selected by overlapping vessel trajectories and aquaculture farm locations. Third, the lateral distance of the vessels from the high-risk area was calculated. Finally, a probability distribution curve was fitted to the acquired lateral distance data, and the separation distance for safe navigation was calculated.

### 2.3. Estimation of the Lateral Distance

In order to estimate the lateral distance between the vessels that navigated in each sea area and the traffic route, we draw a perpendicular line in the direction of the aquaculture farm closest to the traffic route, at the time at which the vessels passed. Then, we extracted the trajectory of the vessel before and after it passes through the perpendicular line in each area. The two trajectories extracted in the above manner were connected by straight lines to obtain the intersection point with the perpendicular line. From this information, the lateral distance was calculated.

### 2.4. Application of the Gaussian Mixture Model (GMM).

The GMM has been widely used in many areas [34,35]. The GMM was applied as the probability distribution in this study because it appeared to be the best-fit probability distribution for the traffic route of vessels. The GMM is known to be more suitable than the normal distribution for straight and curved traffic routes [36]. In this sense, given a collection of data samples x = {$x_{1}$, $x_{2}$, …, $x_{m}$} where each $x_{i}$ represents a random vector, Equation (1) follows, if we assume that x belongs to a *G*-component of a finite mixture distribution [37].

(1) $$p\left(x_{i}|\theta \right)=\;\overset{G}{\underset{j=1}{\sum }}\alpha_{j}p\left(x_{i}|\;\mu_{j},\sigma_{j}\right),\;\;j=1,2,\ldots ,\;G;i=1,2,\ldots ,m$$

subject to: $\sum_{j=1}^{G}\alpha_{j}$ = 1; where $\alpha_{j}$ is the mixing weight, and $\theta_{j}$ is the set of parameters of the *j*-th mixture component $p\left(x_{i}|\mu_{j},\sigma_{j}\right)$. We denote that $\mu_{j}\;$ is the location parameter, and $\sigma_{j}$ is scale parameters of the GMM.

Several approaches exist for estimating the parameters of the GMM, given a set of data points. The most popular, and the one used here, is the well-known Expectation-Maximization (EM) algorithm [38], which iteratively optimizes the model using the Maximum Likelihood Estimates (MLE).

The EM algorithm does not provide any information regarding the selection of the number of mixtures from data. Clearly, such a selection is an important and unavoidable computational issue for GMM and EM. Akaike’s information criterion (AIC) [39] and Bayesian information criterion (BIC) [40] are the two most popular models selection criteria based on penalty terms of the model complexity. AIC and BIC intend to compute the model that best represents the data (i.e., data likelihood term), with a minimum number of free parameters to be estimated (i.e., model complexity term). Unlike the AIC, the BIC penalizes free parameters more strongly. The BIC penalizes model complexity more heavily using Equation (2).

(2) $$BIC=2\log L\left(\hat{\theta }|x\right)-k\log n$$

where *L* is the maximized value of the likelihood function of model, $\hat{\theta }$ are the parameter values that maximize the likelihood function, k is the number of free parameters to be estimated in GMM, and *n* is the number of observations. BIC penalizes models more for free parameters than does AIC. Thus in this paper, we use the BIC for GMM model selection. To use BIC for the best model selection, we simply choose the model that leads to the smallest BIC over the set of possible models.

When the GMM is applied to a continuous random variable *x*, the probability of damage at the location $x_{i}$ is calculated using Equation (3).

(3) $$F_{gmm}\left(x_{i}|\alpha ,\mu ,\sigma \right)=\int_{x_{i}}^{\infty }\left[\overset{G}{\underset{j=1}{\sum }}\frac{\alpha_{j}}{\sigma_{j}\sqrt{2\pi }}exp\left\{-\frac{1}{2}{\left(\frac{x_{i}-\mu_{j}}{\sigma_{j}}\right)}^{2}\right\}\right]dx$$

where $F_{gmm}$ is the cumulative distribution function of the GMM, and $x_{i}$ is the lateral distance from the traffic route to the aquaculture farms.

### 2.5. Estimation of the Goodness-of-Fit Using the Kolmogorov–Smirnov Test

The Kolmogorov–Smirnov (KS) test, which is widely used as a goodness-of-fit test, was conducted to determine whether the GMM follows the data of this study. The KS test analyzes the null hypothesis ($H_{0}$) by comparing the cumulative distribution function (CDF) of the assumed probability distribution with the empirical cumulative distribution function (ECDF) of the sample data. If the *p*-value is less than the significance level, then we reject the null hypothesis and conclude that the GMM does not fit the data well.

$H_{0}$: The data follow a specified probability distribution.

$H_{1}$: The data do not follow a specified probability distribution.

The KS test finds the largest distance between the ECDF and CDF over the entire range of a variable, employing Equation (4) [41].

(4) KSv=|Fe(xi)−Fa(xi)|xsup

where $\;sup_{x}$ is the supremum of the set of probability density distances, $KS_{v}$ is the value of KS, $F_{e}$ is the ECDF, and $F_{a}$ is the assumed CDF.

### 2.6. Estimation of the Annual Frequency of Aquaculture Farm Damages

The annual frequency of aquaculture farm damage was calculated using Equation (5) [42].

(5) $$A_{f}=N\times P_{r}$$

where $A_{f}$ is the annual frequency of aquaculture farm damage, *N* is the annual number of navigating vessels, and $P_{r}$ is the probability of the course of vessels.

Since there is not an internationally accepted standard for the acceptance of risks applicable to navigational safety issues, we employed a criterion suggested by the German authorities that accept a collision probability with a return period of more than 100 years at offshore wind farms [42]. The risk acceptance criterion used in Germany is shown in Table 1 [42].

### 2.7. Description of the Installation of the Interconnected Location Sensors (IoUT)

**Figure 3:**
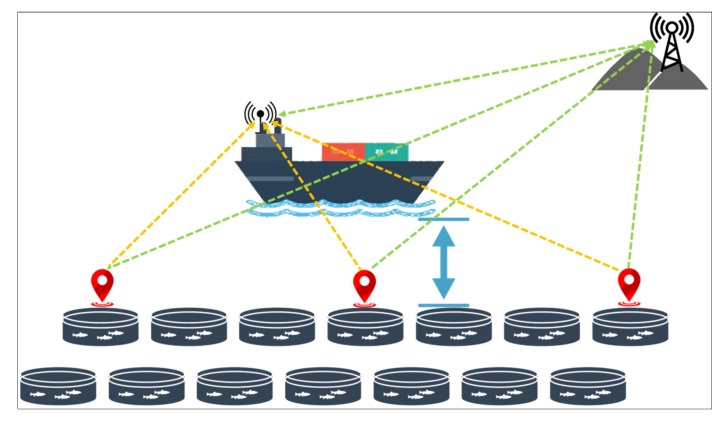
Installation of the interconnected location sensors (IoUT) in the aquaculture farms nearest to the ship traffic route. The orange dotted line indicates the information transmitted to the ship and the green dotted line represents the information transmited to the Long Term Evolution-Maritime (LTE-M) system.

Figure 3 describes the suggestion of installing IoUT on the aquaculture farms closest to the ship traffic route to meet the communication requirements of maritime users in the Republic of South Korea. In this figure, the orange dotted line represents the information transmitted to the ship, and the green dotted line indicates the information transmitted to the Long Term Evolution-Maritime (LTE-M) system using the installed location sensors.

It is worth mentioning that LTE-Maritime is a project that was recently launched in this country with the aim of providing a communication system that supports high data rates of the order of megabits per second in a communication range covering 100 km. The basic idea of the LTE-Maritime project was to apply to the maritime domain the LTE technology currently used in the land region [43,44]. The LTE-Maritime project is a system that uses modern wireless technologies to support and facilitate the communication of ship-to-shore data, anytime and anywhere. Vessels that have received the location information of the aquaculture farms beforehand through the LTE-M system will sail at a safe distance so that they will not cause damage to farm structures.

## 3. Results

### 3.1. Selecting the High-Risk Area

**Figure 4:**
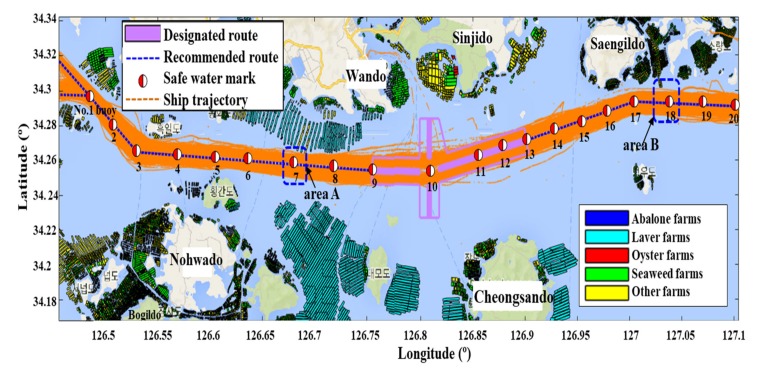
Overlay map with aquaculture farms and vessel trajectories.

In this study, we investigated the aquaculture farms that were adjacent to the traffic route installed to the north of the 7th safe watermark (area A) and the northwest of the 18th safe watermark (area B) as shown in Figure 4. The aquaculture farms in areas A and B are about 3 km away from the coast, in the inshore sea.

This figure illustrates the overlay map with the mentioned aquaculture farms and the vessel trajectories that result from the distribution of these farms and the monthly AIS trajectories of the vessels navigating the traffic route. The distribution of the aquaculture farms is represented by different colors (abalone—blue, laver—oak, oyster—red, seaweed—green, others—yellow).

### 3.2. Lateral Distances in Areas A and B

**Figure 5:**
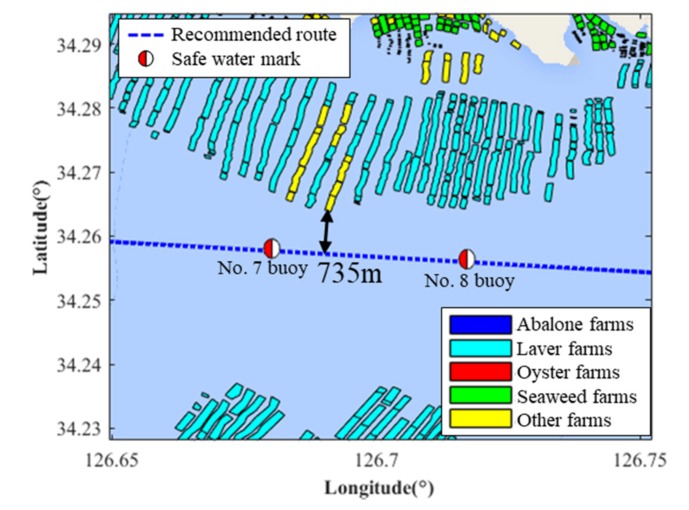
The lateral distance from the traffic route to the closest aquaculture farm in area A.

Figure 5 displays the estimated lateral distances from the traffic route to the closest aquaculture farms A. The lateral distance from the traffic route to the closest aquaculture farm in area A is 735 m. Similarly, the lateral distance from the traffic route to the closest aquaculture farm in area B is 980 m.

### 3.3. Model Selection and Parameters of GMM in Area A and B

The Gaussian mixtures, with *G* = 1, 2, …, 5 components, were fitted to the data. The suitable number of Gausiann components calculated using the BIC are depicted in Table 2 for area A and B.

In addition, Table 3 shows the parameters obtained using the MLE of the GMM fitted to the lateral distance of the navigating vessels in areas A and B. For area A, the first Gaussian component has a scale parameter of 121.6 m and a mixture parameter of 0.45 at a location of 371.4 m. The second Gaussian component has a scale parameter of 122.1 m and a mixture parameter of 0.55 at a location of 406.7 m. For area B, the first Gaussian component has a scale parameter of 98.9 m and a mixture parameter of 0.37 at a location of 307.9 m. The second Gaussian component has a scale parameter of 109.3 m and a mixture parameter of 0.63 at a location of 439.1 m.

**Figure 6:**
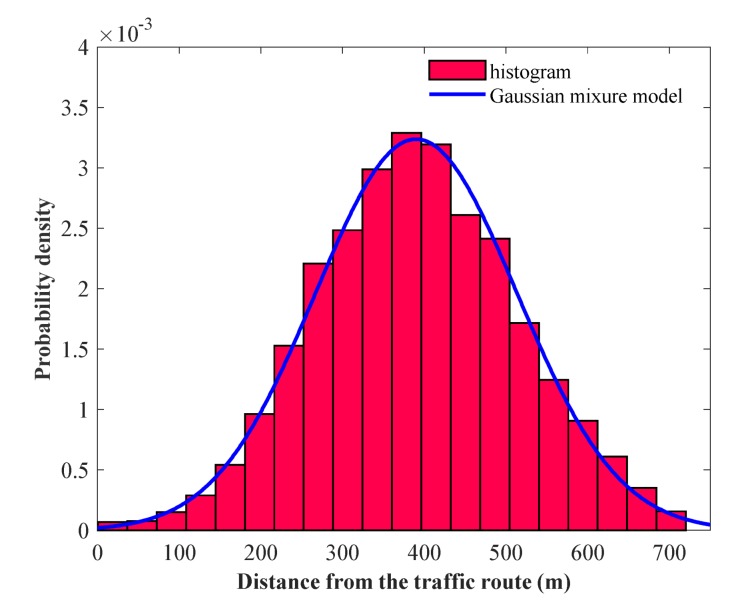
Histogram and Gaussian mixture model for area A.

**Figure 7:**
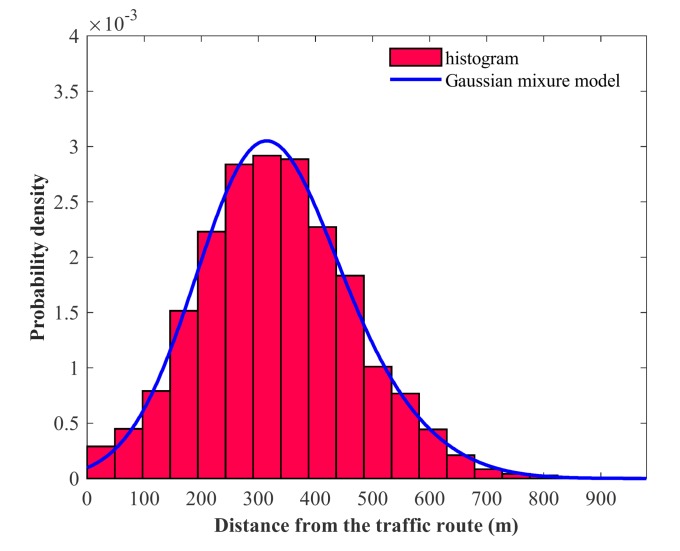
Histogram and Gaussian mixture model for area B.

Finally, Figure 6 and Figure 7 show the histogram and the fitted GMM based on the calculated lateral distance for areas A and B. The horizontal axis represents the lateral distance from the traffic route to the aquaculture farms, and the vertical axis represents the probability density.

### 3.4. Goodness of KS Test in Area A and B

Table 4 shows the statistics of KS test for areas A and B. The KS test statistics for area A computed according to Equation (4) is $KS_{v}$ = 0.01. The KS test statistics for area B is also $KS_{v}$ = 0.01. The threshold values is 0.02 at a significance level of 0.05.

### 3.5. Annual Frequency of Damage and the Return Period for the Aquaculture Farms in Areas A and B.

Table 5 and Table 6 indicate the annual frequency of damage and the return period according to the distance from the traffic route to the aquaculture farms in areas A and B, respectively.

## 4. Discussion

In relation to this research on the estimation of the safe navigation distance between marine routes and the aquaculture farms located in South Korea, it was found that the lateral distances from the traffic route to the nearest aquaculture farm in areas A and B were 735 m and 980 m, respectively.

It is worth mentioning that the results of the KS test for the fitting of the GMM in areas A and B suggested that the GMM is well adjusted to the data $(H_{0}$) in these areas. This outcome is deduced from the fact that in areas A and B, the test results (0.01) were smaller than the threshold (0.02) at a significance level of 0.05; then, since the *p*-values were greater than the level of significance, the null hypothesis cannot be rejected ($H_{0}$).

In addition, it is pertinente to indicate that the Gausiann components shown in Table 2 for areas A and B were calculated using the BIC. In this sense, we selected the optimal number of components from a set of candidate of the GMM. The optimal model was selected based on the minimum BIC. A lower BIC value indicated a better fit. In our experiments, the optimal number of mixture components for every GMM for area A and B were chosen to be two. As the number of Gaussian mixtures increases, we were able to see that there were higher BIC values. We observed that the two-component mixture model was the best model, and used the EM algorithm to estimate the parameters of the two-component mixture model. The samples were initially labeled using the *k*-means++ algorithm [45]. In addition, in Figure 6, it seems like the GMM model follows a normal distribution function, but actually it is a combination of 44% of the first Gaussian component and 55% of the second Gaussian component. In Figure 7, the right-hand side of GMM is a long tail because the mixing weight of the second gaussian component is bigger than that of the first Gaussian component.

Additionally, from the annual frequency of damage and the return period related to the distance from the traffic route to the aquaculture farms in area A, it was deduced that damage occurs 11.4 times per year at the aquaculture farms closest to the traffic route. However, when the aquaculture farms were installed at a distance of 1000 m from the traffic route, the annual frequency of damage was 0.002 and the return period of damage was approximately 601.1 years. Therefore, according to the German criterion mentioned in the Methods section, a safe distance of 1000 m from the traffic route is required to install aquaculture farms in area A. Regarding area B, damage happens at 0.017 times per year at the aquaculture farms closest to the traffic route. However, following the German criterion, when the aquaculture farms were installed a distance of 1000 m from the traffic route, the annual frequency of damage is 0.009 and the return period of damage was approximately 116.0 years. According to these findings, the return period of damage increased by installing aquaculture farms at a minimum distance of 1000 m from traffic routes in the inshore sea.

From these results obtained using the data collected by satellite and aerial images overlapped with the trajectories of vessels navigating along the traffic route, it can be deduced that a distance of at least 1000 m is required to protect the vessels and aquaculture farms.

About this finding, it is convenient to mention that the Maritime New Zealand guidelines proposed that the minimum separation distance between aquaculture farms and traffic routes in the inshore sea should be 500 m [10]. Similarly, other studies related to the general theme of our research, suggested that the minimum distance between wind farms and navigation routes must be 1000 m [46], and 1854 m [47].

Concerning previous studies, the review of the literature indicated that there were no specific published studies on the statistical estimate of the minimum safe distance between aquaculture farms and traffic routes. However, a couple of studies in related fields proposed a mathematical methodology to analyze a problem similar to that investigated in our research [14,15]. Wawruch and Stupak [14] suggested a collision probabilistic model to evaluate the safe distance between wind farms and vessel traffic routes. They modeled the maritime traffic in the coastal area of the Polish maritime waters using a Normal distribution, without performing a fitness test. Since the Normal distribution used by the authors may not be suitable due to the characteristics of the North Sea, other probability distributions might have been more appropriated in the mentioned case. On the other hand, Bekker and Schmid [15] used graph theory to investigate the safe transit of a vessel through a sea mapped minefield. In this study, the sea was assumed as a grid where the graph nodes denote locations, and the edges symbolize the cost of travel between every node. The cost considers the distance between the grid points and the danger linked to the journey between these points. Although this model can be appropriated in some circumstances, the modeling of aquaculture farms in the form of point sources of danger is not practical.

In another context, the area coverage of aquaculture farms increases every year [6]. Therefore, the separation distance between aquaculture farms and traffic routes is decreasing. This is problematic for aquaculture farms located near traffic routes and for the vessels navigating these traffic routes. As a result, navigators must ensure a sufficient separation distance between traffic routes and the nearest aquaculture farms for safe navigation. Navigators require that aquaculture farms be installed at a considerable distance from traffic routes. If the fisheries attempt to install aquaculture farms within 1000 m from traffic routes, then a rigorous application of the law with respect to illegal farms is required. In this scenery, it is necessary to properly plan the installation and management of aquaculture farms using marine spatial planning and an integral management process of all sea resources that ensures the sustainability of fishery installations, security, and the ecology of the marine and coastal spaces [8,9,22,48,49,50,51].

If the farm is located very near to the traffic route within 1000 m, we suggest that it is necessary to install a set of IoUT sensors that can transmit the location of the aquaculture farms to the ships. In this regard, we proposed to install a set of interconnected sensors using the Long Term Evolution-Maritime (LTE-M) system launched in South Korea as explained in the Materials and Methods section. The location of the farms will be communicated wirelessly to the ship’s navigator to avoid damage to the fish farms. In the field of IoUT, there is not much research on the transmission of the location of information from farms to ships. However, as the aquaculture is one of the fastest-growing food-producing industries in the world, more research in this field is needed for the protection of the aquaculture farms and the safety of ships.

## 5. Conclusions

In this study, the aquaculture farm data collected by satellite and aerial images were overlapped with the trajectories of the vessels navigating along the marine traffic route. Then, a high-risk area in South Korea was selected through the overlapping, and the annual frequency of damage was calculated based on a probability distribution curve applied to the area. From this analysis, the appropriate distance between aquaculture farms and the traffic route was derived. In particular, the GMM was applied, and the KS test was used. It was found that a safe distance of at least 1000 m is needed to protect farms and vessels.

This proposed method of calculating the minimum separation distance between the traffic routes and aquaculture farms suggested that it is possible to prevent damage to vessel propellers and fisheries locating aquaculture farms at the minimum safe distance from the traffic routes. If the aquaculture farms are located within 1000 m, from the route of the ships we recommemd that it is necessary to install a set of interconnected IoUT sensors that can transmit the location of the aquaculture farms to the ships to avoid damage to the vessels propellers and the structure of the farms.

In this study, the separation distances were analyzed based solely on vessel trajectories. The surveys of shipmasters and navigators will be considered in a future study. The importance of this study is that it is a robust statistical method that can be applied to a variety of similar situations and can be useful in marine spatial planning related to the installation and new developments of aquaculture farms. In addition, the suggested method can provide guidelines for future policies and laws related to protection or insurance topics related to farm placing.

## Figures and Tables

**Table 1:** Risk acceptance criteria in Germany (based on data [42]).

Acceptance	The Time Between Collisions (Years)
Acceptable	> 100
Further analysis necessary	50–100
Not acceptable	< 50

**Table 2:** Bayesian information criterion (BIC) values according to the number of mixture components for areas A and B.

Number of Mixture Components	BIC for Area A	BIC for Area B
2	55,115	55,803
3	55,129	55,821
4	55,163	55,844
5	55,186	55,858

**Table 3:** Parameters estimated by Maximum Likelihood Estimates (MLE) of the Gaussian mixture model for area A and B.

	Parameters of GMM for Area A	Parameters of GMM for Area B
Location parameter	$\mu_{1}$ = 371.4	$\mu_{1}$ = 307.9
	$\mu_{2}$ = 406.7	$\mu_{2}$ = 439.1
Scale parameter	$\sigma_{1}$ = 121.6	$\sigma_{1}$ = 98.9
	$\sigma_{2}$ = 122.1	$\sigma_{2}$ = 109.3
Mixture parameter	$\alpha_{1}$ = 0.45	$\alpha_{1}$ = 0.37
	$\alpha_{2}$ = 0.55	$\alpha_{2}$ = 0.63

**Table 4:** Statistics of Kolmogorov–Smirnov test for area A and B.

	$KS_{v}$	Critical Value	*p*-Value	Significance Level
area A	0.01	0.02	0.75	0.05
area B	0.01	0.02	0.78	0.05

**Table 5:** Analysis of the annual frequency of damage according to the distance from the traffic route to the aquaculture farms in area A.

Distance from the Traffic Route to the Aquaculture Farms	Probability	Annual Frequency of Damage	Return Period
735 m	2.59 × 10^−3^	11.4	0.1 years
800 m	4.45 × 10^−4^	1.9	0.5 years
850 m	9.61 × 10^−5^	0.4	2.4 years
900 m	1.77 × 10^−5^	0.07	12.8 years
950 m	2.80 × 10^−6^	0.01	80.9 years
1000 m	3.76 × 10^−7^	0.002	601.1 years

**Table 6:** Analysis of the annual frequency of damage according to the distance from the traffic route to the aquaculture farms in area B.

Distance from the Traffic Route to the Aquaculture Farms	Probability	Annual Frequency of Damage	Return Period
980 m	3.81 × 10^−6^	0.017	59.5 years
1000 m	1.95 × 10^−6^	0.009	116.0 years
